# Clinical outcomes of ablation of gastric dysplasia with argon plasma coagulation

**DOI:** 10.1371/journal.pone.0306934

**Published:** 2024-07-09

**Authors:** Mi Jin Oh, Sang Gyun Kim, Jiyoon Kim, Yun Suk Na, Seunghan Lee, Junhee Lee, Bokyung Kim, Hyunsoo Chung, Soo-Jeong Cho

**Affiliations:** Department of Internal Medicine and Liver Research Institute, Seoul National University College of Medicine, Seoul, Republic of Korea; PLOS ONE, UNITED KINGDOM

## Abstract

**Background:**

Although several small cohort studies have shown the utility of argon plasma coagulation (APC) in the treatment of gastric dysplasia, its clinical significance has not been established. This study aims to assess the efficacy of APC as a first line treatment for gastric dysplasia, and identify risk factors for residual dysplasia.

**Methods:**

A total of 179 cases of gastric dysplasia were treated with APC and have been followed-up with upper endoscopy within 1 year. The overall incidence and the characteristics of lesions with residual dysplasia in follow-up endoscopy were analyzed by logistic regression.

**Results:**

Among 179 lesions treated with APC, 171 (95.5%) lesions have achieved complete ablation in the follow-up endoscopy. Additional APC was applied for residual dysplasia, achieving complete ablation in 97.77% (175/179). The upper third location of the gastric dysplasia was significantly associated with residual dysplasia, while tumor size, horizontal location, macroscopic morphology and grade of dysplasia showed no significant associations with residual dysplasia following the initial APC.

**Conclusions:**

APC with meticulous follow-up can be recommended as a first line treatment in patients with gastric dysplasia.

## Introduction

Gastric dysplasia is a well-known precursor of gastric cancer. It can be largely classified into high and low-grade dysplasia according to the histologic finding. Previous studies have shown that 0–20% of low-grade and 60–85% of high-grade dysplasia can progress to gastric cancer [1–4]. Most cases of low-grade dysplasia remain as stable forms without progression to invasive cancer over time.

Currently, endoscopic resection has been recommended for the treatment of gastric dysplasia in most guidelines [5, 6] as it allows *en bloc* resection and accurate pathologic diagnosis. However, this procedure requires more resources including skilled professionals, and can lead to serious complications such as bleeding and perforation [7]. Even after a complete resection, endoscopic surveillance is still needed for detection of synchronous or metachronous lesions [8].

Argon plasma coagulation (APC) is a non-contact method of electrocoagulation using ionized argon gas. Recently, APC has been applied in various endoscopic procedures, including hemostasis of vascular lesions and ablation of malignant or premalignant lesions [9–11]. In studies comparing the outcomes of APC and endoscopic submucosal dissection for the treatment of gastric neoplasms, the local recurrence rate was found to be higher in the APC group [12–15]. However, most of the recurred lesions were completely ablated through additional APC, and the complication rate was minimal. Moreover, the overall cost was lower, and the time needed for treatment was significantly shorter in the APC group [12–15]. Thus, APC can be a safe alternative for the primary treatment of gastric dysplasia. However, since most studies included only a limited number of patients, with the largest cohort including only 116 lesions who had received APC for gastric dysplasia, its clinical significance and risk factors for incomplete ablation has not been fully established. In this study we retrospectively reviewed a large cohort of gastric dysplasia lesions that have been treated with APC as a first line treatment, in order to assess the curative ablation rate of APC and identify risk factors for residual dysplasia.

## Materials and methods

### Patient selection and definitions

Medical records of patients treated with APC for gastric dysplasia with a curative intent at Seoul National University Hospital between 1^st^ of January, 2020 and 30^th^ of April, 2021 were retrospectively reviewed between 23^rd^ of May, 2022 and 22^nd^ of May, 2023. Lesions that locally recurred at the sites of previous endoscopic resection or APC were excluded. Patients were primarily followed-up at 1 year after the APC procedure with an endoscopic exam for the presence of residual lesions or synchronous/metachronous recurrences, but the follow-up duration varied according to the characteristics of the initial tumor. In follow-up endoscopy, the ablation site was carefully examined. Lesions with residual dysplasia at the APC site in follow-up endoscopy was defined as “residual dysplasia” group, and was confirmed by pathologic examination. Completely ablated lesions without residual dysplasia in follow-up endoscopy were defined as no residual dysplasia” group. Some of the patients within the “residual dysplasia” group were treated with additional APC, and those who have achieved curative ablation with repeated APC were defined as the “final curative ablation” group.

Vertical location of the lesion was divided into three sections of the stomach: upper, middle, and lower thirds. Horizontal location was divided into four: anterior wall, lesser curvature, posterior wall, and greater curvature. Gross morphology of the tumor was categorized into elevated, flat, and depressed, according to the Paris classification [16].

This study conducted in accordance with the Declaration of Helsinki and was approved by the Institutional Review Board of the Seoul National University Hospital (IRB No. 2205-068-1323). The requirement for informed consent was waived due to its retrospective nature. The collected data were anonymized before analysis.

### APC procedure

APC was performed using pulsed APC mode on VIO 300D (Erbe elektromedizin GmbH, Tubingen, Germany), with argon gas flow rate set at 1.8L/min, max power at 40W and effect at 2. Targeted lesions were treated by APC until the whole lesion appeared dry and gray-brown on the endoscopic examination.

### Statistical analysis

Baseline characteristics of the residual dysplasia group and no residual dysplasia group were compared. Continuous variables were represented as median values [interquartile range] and were compared using Mann-Whitney U test, while categorical variables were represented as proportions and were compared using Fisher’s exact test. Univariate logistic regression model for the presence of a residual dysplasia included variables that differed significantly between the two groups or held clinical importance. Variables that demonstrated statistical significance (p<0.1) in univariate model were considered eligible for multivariate logistic regression model.

All statistical analyses were performed using R version 4.3.0 (R Core Team, 2023). Statistical significance was defined as a two-sided p value < 0.05.

## Results

### Baseline characteristics

**Fig 1:**
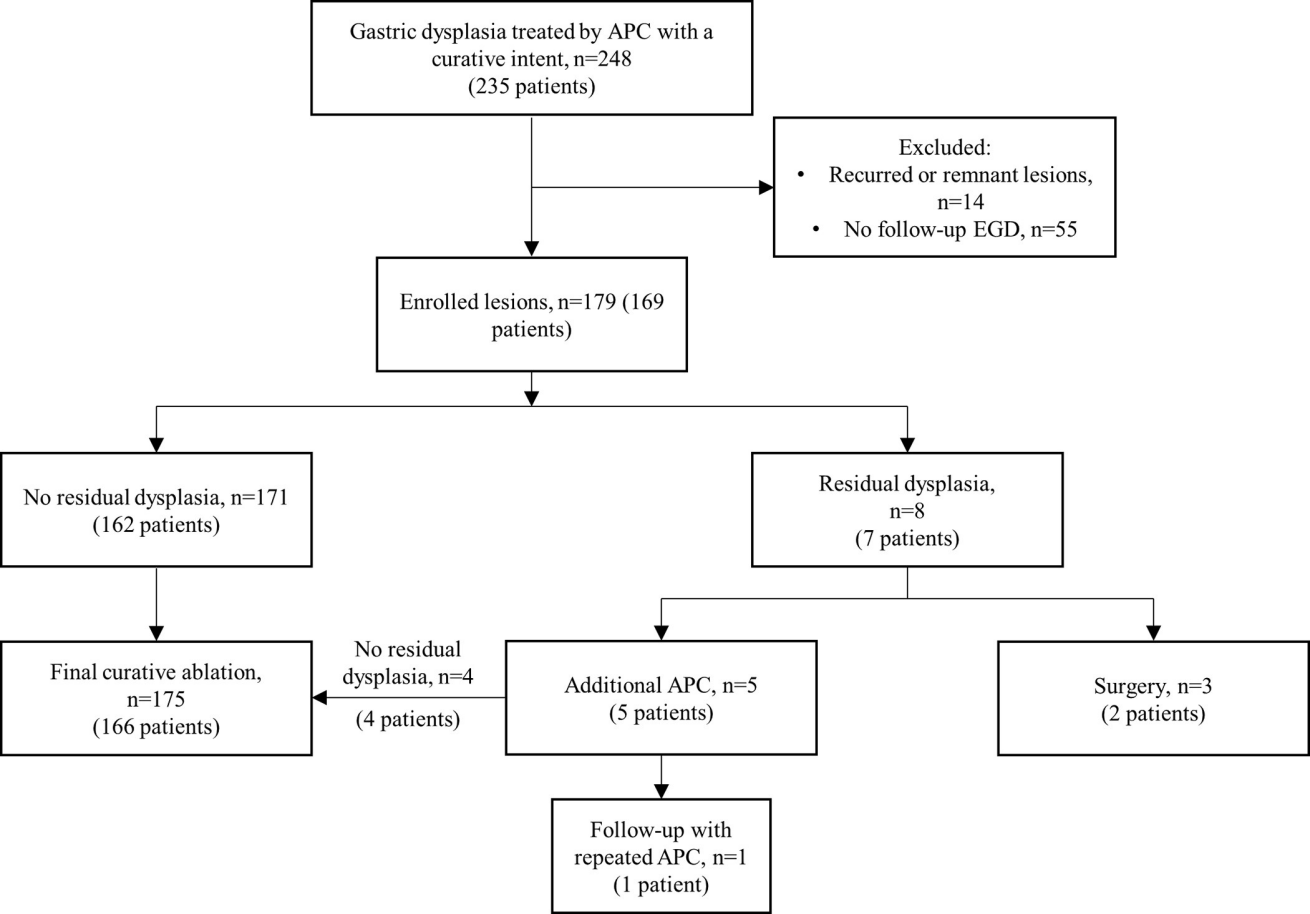
Flow chart of the study showing patient enrollment and profiles of patients with residual dysplasia.

A total of 248 gastric dysplasia lesions in 235 patients were treated with APC with a curative intent in Seoul National University Hospital between January 2020 and April 2021. The lesions treated with APC were mostly smaller than 1.5cm regardless of pathologic grade. Among the 248 lesions, 14 lesions that had locally recurred after previous endoscopic resection, and 55 lesions that lacked follow-up endoscopy were excluded. Ultimately, 179 lesions in 169 patients were included for analysis (Fig 1).

Among the 179 lesions, 171 lesions had no residual dysplasia (no residual dysplasia group), while 8 lesions had residual dysplasia in the follow-up endoscopy after a single session of APC (residual dysplasia group). In baseline characteristics, the two groups showed no significant difference in age and sex (Table 1). Regarding clinicopathological characteristics, specifically in terms of vertical location of the initial dysplasia, the residual dysplasia group had a higher proportion of tumors located in the upper part of the stomach, while the no residual dysplasia group had more tumors located in the middle and lower part of the stomach (p-value = 0.004). In terms of the initial tumor size, the proportion of tumors larger than 10mm was higher in the residual dysplasia group, while the proportion of tumors smaller than 10mm were higher in the no residual dysplasia group (p-value 0.022). On the other hand, there were no significant differences between the two groups in terms of horizontal location, macroscopic morphology and grade of dysplasia.

**Table 1:** Baseline characteristics of the residual dysplasia group and no residual dysplasia group.

	After single-session APC		p-value
	Residual dysplasia (n = 8)	No residual dysplasia (n = 171)	
Male (%)	6 (75)	113 (66.1)	0.720
Age (years)	69.0 [67.0–72.0]	70.0 [64.5–78.0]	0.683
Vertical location of tumor (%)			0.004
Upper	3 (37.5)	4 (2.3)	
Middle	1 (12.5)	61 (35.7)	
Lower	4 (50.0)	101 (59.1)	
Remnant stomach	0 (0.0)	5 (2.9)	
Horizontal location of tumor (%)			0.910
Anterior wall	1 (12.5)	31 (18.1)	
Lesser curvature	4 (50.0)	64 (37.4)	
Posterior wall	1 (12.5)	37 (21.6)	
Greater curvature	2 (25.0)	24 (19.9)	
Remnant stomach	0 (0.0)	5 (2.9)	
Size of tumor			0.022
≤10mm	4 (50.0)	141 (82.5)	
>10mm and ≤20mm	3 (37.5)	28 (16.4)	
>20mm	1 (12.5)	2 (1.2)	
Macroscopic morphology			0.558
Elevated	4 (50.0)	112 (65.5)	
Flat	4 (50.0)	54 (31.6)	
Depressed	0 (0.0)	5 (2.9)	
Pathology of tumor			0.486
Low-grade dysplasia	7 (87.5)	158 (92.4)	
High-grade dysplasia	1 (12.5)	13 (7.6)	

### Risk factors for residual dysplasia after a single-session of APC

Factors associated with residual dysplasia after a single-session of APC are shown in Table 2. In the univariate logistic regression analysis, vertical location and tumor size were significantly associated with the presence of residual dysplasia. Tumors located in the middle [Odds ratio (OR) for residual dysplasia 0.02; 95% confidence interval (CI) 0.00–0.26; p-value = 0.0025] or lower [OR 0.05; 95% CI 0.01–0.32; p-value 0.0014] third of the stomach was significantly associated with a lower risk of residual dysplasia compared to tumors located in the upper third of the stomach. Tumors larger than 20mm [OR 17.62; 95% CI 1.31–236.83; p-value 0.0304] were significantly associated with a higher risk of residual dysplasia compared to tumors smaller than 10mm. Tumors between 10mm and 20mm showed a tendency towards a higher risk of residual dysplasia without statistical significance [OR 3.78; 95% CI 0.80–17.81;p-value 0.0931]. Other clinicopathological factors of the initial dysplasia such as the horizontal location, macroscopic morphology and pathologic grade showed no significant association with the presence of a residual dysplasia (Table 2).

**Table 2:** Risk factors of residual dysplasia after a single-session of APC ablation.

	Univariate logistic regression			Multivariate logistic regression		
	Odds ratio	95% CI	p-value	Odds ratio	95% CI	p-value
Vertical location						
Upper	1			1		
Middle	0.02	0.00–0.26	0.0025	0.02	0.00–0.33	0.0052
Lower	0.05	0.01–0.32	0.0014	0.08	0.01–0.59	0.0127
Horizontal location						
Anterior wall	1					
Lesser curvature	1.94	0.21–18.07	0.5615			
Posterior wall	0.84	0.05–13.95	0.9019			
Greater curvature	1.82	0.16–21.12	0.6307			
Size of tumor						
≤10mm	1			1		
>10mm and ≤20mm	3.78	0.80–17.81	0.0931	3.70	0.69–19.88	0.1273
>20mm	17.62	1.31–236.83	0.0304	12.01	0.27–532.79	0.1988
Macroscopic morphology						
Elevated	1					
Flat	2.07	0.50–8.61	0.3151			
Depressed	0					
Pathology of tumor						
Low-grade dysplasia	1					
High-grade dysplasia	1.74	0.20–15.21	0.6183			

The vertical location and tumor size were included in the multivariate logistic regression model, and only the vertical location was significantly associated with the risk of residual dysplasia (middle third [OR 0.02; 95% CI 0.00–0.33; p-value 0.0052], lower third [OR 0.08; 95% CI 0.01–0.59; p-value 0.0127]). Although the larger tumor size showed a trend towards an increased risk of residual dysplasia, it did not reach statistical significance (Table 2).

### Clinical outcomes of gastric dysplasia treated with APC

In clinical outcomes, the median follow-up duration of the lesions were 363 days (Table 3). In 171 (95.5%) out of 179 lesions, complete ablation was achieved without any residual dysplasia in the follow up endoscopy. Among the 8 lesions with residual dysplasia, 5 were treated with additional APCs, and 4 were ultimately cured by the second APC treatment (Table 4). Thus, 175 (97.77%) out of the 179 lesions achieved curative ablation by follow-up with additional APC. In one case, residual dysplasia was still noted after the second APC treatment. This case received two additional rounds of APC, and the patient is currently under clinical follow-up. In this particular case, the initial dysplasia was 3cm in size and located in the cardia. Three lesions in the residual dysplasia group were treated with surgery, one of which a metachronous gastric cancer were detected in the follow-up endoscopy (Fig 1). Two residual cases were synchronous low-grade gastric dysplasia lesions in a single patient, located in the cardia and antrum. The lesion in the gastric cardia progressed to high-grade dysplasia in the 1 year follow-up endoscopy. Considering the technical difficulty of endoscopic resection in lesions in the cardia with underlying fibrotic scars, the patient was referred for surgery.

**Table 3:** Short-term outcomes of APC.

Tumors, n = 179	
Follow up period, median (range) (days)	363 (160–622)
No residual dysplasia (%)	171 (95.5)
Final curative ablation by repeated APC (%)	175 (97.77)
Complication (%)	4 (2.23)
Bleeding	3 (1.68)
Perforation	0
Others	1 (0.56)
Metachronous neoplasm (%)	7 (3.91)

**Table 4:** Characteristics of the residual lesions, further treatment sequence and outcomes.

Study No.	Sex	Age	Pathology of the initial lesion	Time of recurrence after APC (months)	Pathologyof the recurred lesion	Second treatment	Follow-up duration after second treatment (months)	Recurrence at last follow-up
15^a^	M	68	LGD	6.4	LGD	Surgery	12.0	No
16^a^	M	68	LGD	6.4	HGD	Surgery	12.0	No
18	M	64	LGD	12.3	LGD	APC	12.0	No
51	M	73	LGD	12.1	LGD	APC	18.9	No
65	F	70	LGD	12.3	LGD	APC	6.0	No
114	M	66	LGD	5.3	LGD	APC	19.1	No
138	F	77	LGD	12.1	LGD	Surgery^b^	12.7	No
168	M	71	HGD	9.5	LGD	APC	12.1	Yes

F, Female; M, Male; LGD, low-grade dysplasia; HGD, high-grade dysplasia; APC, argon plasma coagulation

^a^ Subject No. 15 and 16 are two synchronous lesions in one patient.

^b^ Subject No. 138 had residual low grade dysplasia but receieved surgery due to a metachronous cancer.

Among the 179 lesions, seven lesions (3.91%) in six patients were accompanied by metachronous neoplasms during follow-up beyond 1 year (S1 Table). Of the six patients who developed a metachronous neoplasm, three were diagnosed with gastric cancer, one with high-grade dysplasia, and two with low-grade dysplasia. Among the gastric cancer cases, one was treated by endoscopic submucosal dissection (ESD), while two underwent surgery. All cases of metachronous gastric dysplasia were treated with additional APC.

Only four cases (2.23%) experienced complications following the APC procedure. Among these, three (1.68%) cases presented with delayed bleeding between 7–12 days after the procedure, and one case (0.56%) reported abdominal discomfort. No lesions were associated with perforation.

## Discussion

In recent years, the application of APC has extended to a wide range of gastrointestinal disorders, notably including the ablation of gastric neoplasms. Previous studies have also showed the effectiveness of APC for the patients with early gastric cancer (EGC) who could not withstand surgery or endoscopic resection [17–19], as well as for residual tumors after endoscopic resection for EGC [11]. Compared to endoscopic resection, APC is a less expensive, safer and simpler procedure [13–15]. For clinicians, it is easier to learn and requires less time and effort. One study demonstrated that the outcomes of APC in the treatment of EGC were equal in both experienced and non-experienced endoscopists [19]. However, only a handful of studies have shown the clinical outcomes of APC in gastric dysplasia, which involved cohorts of gastric neoplasms, ranging from 65 to 320 lesions, which received either endoscopic resection or APC. Moreover, the actual number of lesions that received APC were limited (with the largest cohort including 116 low-grade gastric dysplasia lesions), with some studies also including early gastric cancer lesions [12–15, 20]. Consequently, the aim of this study was to evaluate the risk of residual lesions within a larger cohort comprising exclusively gastric dysplasia lesions. Furthermore, we sought to include gastric dysplasia lesions regardless of histologic grade, size, and location, in order to identify specific clinical risk factors for residual lesions following APC. Consequently, we included a total of 248 gastric dysplasia lesions treated with APC with a curative intent between January 2020 and April 2021. After the exclusion of lesions lacking follow-up endoscopy, 179 lesions were included for analysis. To the best of our knowledge, this represents the largest cohort to date comprising solely gastric dysplasia lesions treated with APC as a primary treatment modality.

In studies regarding the use of APC in the ablation of gastric dysplasia, the short-term local recurrence rate ranged from 1.7–15.3% [12–15, 20]. In our study, 8 cases (4.5%) had residual dysplasia in the 1-year follow-up endoscopy. Among these 8 cases, 3 out of 4 cases that received additional APC achieved curative ablation. Among the cases in the residual dysplasia group, 3 underwent surgery, with one case due to metachronous gastric cancer discovered during follow-up endoscopy. In effect, only 3 lesions (1.5%) required repeated APC or surgery due to local recurrence even after 1 year. This rate is comparable to the local recurrence rate observed in gastric dysplasia treated by ESD, which ranged from 0% to 3.5% in previous studies [13–15]. Considering that long-term endoscopic surveillance is needed for synchronous and metachronous recurrence after endoscopic resection [8], APC with careful surveillance can serve as an alternative treatment option for the treatment of gastric dysplasia.

Previous studies have failed to identify the risk factors of residual dysplasia or local recurrence due to the limited number of patients who received APC as a first line treatment. One study indicated that low power setting (40W) and non-lifting were risk factors for local recurrence, though these factors are only relevant during the APC procedure [12]. In our study, the vertical location of the tumor showed a significant association with residual dysplasia in both multivariate and univariate analyses. This may be attributed to the challenges in visualizing and accessing lesions of the cardia and fundus using an endoscope. Remarkably, the histologic differentiation of the lesion did not confer a significant risk for incomplete ablation. Regarding the tumor size, large tumors were associated with a higher risk of residual dysplasia in the univariate analysis, it did not achieve statistical significance in the multivariate analysis. Overall, this suggests that gastric dysplasia located in the middle or lower-third of the stomach can be a more suitable candidate for APC.

According to the literature, the complication rate of APC has been reported to be 0–4.7%, with only a few cases of pneumoperitoneum, and mostly minor delayed bleeding events [12–15, 20]. In line with these results, there were only four cases that experienced procedure-related complications in our study, of which three cases were delayed bleeding and one was abdominal discomfort. Serious complications including massive bleeding or perforation did not occur in our study.

In this study, many patients were referred out after the 1-year follow-up endoscopy, and the surveillance schedule varied greatly between patients. Despite this bias, 3.5% of the patients included in our study experienced metachronous recurrence, which is comparable to the metachronous recurrence rate reported in previous studies with a mean follow-up duration of 1–3 years [21, 22]. This data underscores the importance of annual surveillance after APC, similar to endoscopic resection.

This study has several limitations related to its retrospective design. Although our study included lesions with high-grade dysplasia and lesions larger than 2cm, the majority of lesions were of low-grade and smaller than 2cm, which may have led to a selection bias. In fact, most previous studies excluded high-grade lesions and lesions larger than 2cm [13, 14, 20]. Hence, further validation is warranted for the application of APC in these particular cases. Furthermore, due to the low rate of incomplete ablation, the results of the logistic regression model showed low statistical power. Nevertheless, this study has identified that the vertical location of the tumor was significantly associated with incomplete ablation. This can assist clinicians in selecting gastric dysplasia lesions that might respond well to APC treatments. Another limitation of this study is the short follow-up time. As mentioned above, longer follow-up is needed to explore the long-term outcomes including the local and metachronous recurrence rate beyond the first year and the rate of progression to cancer.

In conclusion, APC can be considered as an alternative primary treatment option for gastric dysplasia, especially in low-grade lesions, smaller than 20mm. Particularly, tumors located in the upper third of the stomach showed a significant association with residual tumors, suggesting that tumors in the middle or lower thirds may be more suitable for APC ablation. Same as in cases treated with endoscopic resection, meticulous follow-up is needed after APC for gastric dysplasia to detect of residual, synchronous or metachronous tumor development.

## Supporting information

S1 Dataset Deidentified original dataset. (XLSX)

S1 Table Characteristics of metachronous gastric neoplasms and its treatment sequence. (DOCX)
