# Supplementary material for: Clinical outcomes of ablation of gastric dysplasia with argon plasma coagulation
Source: PLoS One. 2024 Jul 9;19(7):e0306934. doi: 10.1371/journal.pone.0306934 (PMC11232966; doi:10.1371/journal.pone.0306934)
Supplement: S1 Table — (DOCX) [file pone.0306934.s002.docx]

**S1 Table. Characteristics of metachronous gastric neoplasms and its treatment sequence**

| **Study No.** | **Sex** | **Age** | **Pathology of the initial tumor** | **Time of metachronous recurrence after APC (months)** | **Pathology of the recurred lesion** | **Treatment for metachronous lesions** |
| --- | --- | --- | --- | --- | --- | --- |
| 5^a^ | F | 66 | Low-grade dysplasia | 35.2 | Tubular adenocarcinoma, moderately differentiated | ESD |
| 6^a^ | F | 66 | Low-grade dysplasia | 35.2 | Tubular adenocarcinoma, moderately differentiated | ESD |
| 48 | M | 69 | Low-grade dysplasia | 24.7 | High-grade dysplasia | APC |
| 50 | F | 75 | Low-grade dysplasia | 24.3 | Low-grade dysplasia | APC |
| 91 | M | 78 | Low-grade dysplasia | 13.5 | Low-grade dysplasia | APC |
| 138 | F | 77 | Low-grade dysplasia | 12.1 | Tubular adenocarcinoma, well differentiated | Surgery |
| 160 | M | 86 | Low-grade dysplasia | 14.7 | Tubular adenocarcinoma, moderately differentiated | Surgery |

F, Female; M, Male; ESD, endoscopic submucosal dissection; APC, argon plasma coagulation

^a^ Subject No. 5 and 6 are two synchronous lesions in one patient.
